# In-House Fabrication and Validation of 3D-Printed Custom-Made Medical Devices for Planning and Simulation of Peripheral Endovascular Therapies

**DOI:** 10.3390/diagnostics15010008

**Published:** 2024-12-25

**Authors:** Arianna Mersanne, Ruben Foresti, Chiara Martini, Cristina Caffarra Malvezzi, Giulia Rossi, Anna Fornasari, Massimo De Filippo, Antonio Freyrie, Paolo Perini

**Affiliations:** 1 Vascular Surgery, Cardio-Thoracic and Vascular Department, University-Hospital of Parma, 43126 Parma, Italy; 2 Department of Medicine and Surgery, University of Parma, Via Gramsci 14, 43126 Parma, Italy; ruben.foresti@unipr.it (R.F.);; 3 Center of Excellence for Toxicological Research (CERT), University of Parma, 43126 Parma, Italy; 4 Italian National Research Council, Institute of Materials for Electronics and Magnetism (CNR-IMEM), 43124 Parma, Italy; 5 Diagnostic Department, University-Hospital of Parma, Via Gramsci 14, 43126 Parma, Italy; 6 Department of Medicine and Surgery, Section of Radiology, University of Parma, Maggiore Hospital, Via Gramsci 14, 43126 Parma, Italy

**Keywords:** 3D printing, surgical simulation, planning, training, endovascular surgery, vascular surgery, custom-made medical device, patient-specific, 3D-printed vascular model

## Abstract

**Objectives:** This study aims to develop and validate a standardized methodology for creating high-fidelity, custom-made, patient-specific 3D-printed vascular models that serve as tools for preoperative planning and training in the endovascular treatment of peripheral artery disease (PAD). **Methods:** Ten custom-made 3D-printed vascular models were produced using computed tomography angiography (CTA) scans of ten patients diagnosed with PAD. CTA images were analyzed using Syngo.via by a specialist to formulate a medical prescription that guided the model’s creation. The CTA data were then processed in OsiriX MD to generate the .STL file, which is further refined in a Meshmixer. Stereolithography (SLA) 3D printing technology was employed, utilizing either flexible or rigid materials. The dimensional accuracy of the models was evaluated by comparing their CT scan images with the corresponding patient data, using OsiriX MD. Additionally, both flexible and rigid models were evaluated by eight vascular surgeons during simulations in an in-house-designed setup, assessing both the technical aspects and operator perceptions of the simulation. **Results:** Each model took approximately 21.5 h to fabricate, costing €140 for flexible and €165 for rigid materials. Bland–Alman plots revealed a strong agreement between the 3D models and patient anatomy, with outliers ranging from 4.3% to 6.9%. Simulations showed that rigid models performed better in guidewire navigation and catheter stability, while flexible models offered improved transparency and lesion treatment. Surgeons confirmed the models’ realism and utility. **Conclusions:** The study highlights the cost-efficient, high-fidelity production of 3D-printed vascular models, emphasizing their potential to enhance training and planning in endovascular surgery.

## 1. Introduction

Peripheral balloon angioplasty is a widely utilized, minimally invasive procedure in endovascular surgery for treating peripheral arterial disease (PAD) [1,2,3]. While advancements in medical technology have significantly improved the success rates of these procedures, there remains a constant quest for innovative approaches to enhance procedural planning, training, and simulation [4,5,6]. The importance of simulation is reiterated by the newest guidelines of the European Society of Vascular and Endovascular Surgery, which recommend that the vascular surgery training curriculum should include simulation-based training in open and endovascular procedures [3]. Preoperative planning traditionally relies on two-dimensional images, which lack depth information, hindering the understanding of spatial relationships and the evaluation of device fitting and tissue response [7]. Despite the growing use of high-fidelity and mixed reality (VR/MR) simulators for preoperative, patient-specific simulations, they are often unavailable due to substantial financial costs [8,9,10,11]. Alternatively, cadaveric simulation provides an option for endovascular training, particularly for developing tactile skills, though it can also be expensive or impractical [10,12]. In this context, simulation-based training and planning with patient-specific 3D-printed models is emerging as a promising approach, offering a low-cost, patient-specific alternative for endovascular training that warrants further exploration [8,9,10,13,14].

The literature has shown that 3D-printed models can be produced in-house at low-cost and within reasonable timeframes, making them a cost-effective option for resident training and a tailored solution for pre-procedural planning [13]. Training with 3D-printed models has been shown to increase self-confidence and technical skills among trainees, while prompting changes in preoperative planning, particularly in complex endovascular procedures, and for modifying custom-made grafts [15,16]. These models have also proven to be helpful in reducing the dose of contrast medium, as well as fluoroscopy and operating time [16,17]. By accurately replicating anatomical structures, 3D-printed models allow for simulating procedural scenarios, testing, and selecting the most appropriate surgical instruments, potentially enhancing the proficiency of medical personnel, and ultimately leading to improved patient outcomes and safety [13,15,18].

In this context, the accuracy of patient-specific 3D-printed models is crucial for precise interpretation of spatial relationships and evidence-based decision-making. However, existing studies on these models demonstrate substantial variability in the materials and methods employed for their fabrication, with many studies asserting high-fidelity without the robust quantitative analysis to support them [13]. Cumulative errors can arise at various stages, ranging from scan settings and image quality to processing and post-processing techniques, potentially affecting the final printed model [19,20]. Such variations and potential inaccuracies highlight the need for standardized protocols and rigorous validation of model fidelity to ensure that these printed representations accurately reflect patient anatomy. Finally, custom-made 3D-printed models intended for medical use should be classified as medical devices and, consequently, must adhere to quality assurance requirements stipulated by the European Union Medical Devices Regulation (EU MDR 2017/745) [21]. However, current studies in the literature rarely address the use of these models in accordance with these regulations [20]. Therefore, despite their increasing application in vascular surgery for planning and educational purposes, there remains a noticeable gap in the literature concerning their regulatory and manufacturing aspects.

The aim of this study is to describe a standardized methodology for creating in-house, custom-made, 3D-printed models, according to EU MDR 2017/745. Evaluation of the generated models includes both a dimensional accuracy assessment and a performance in real-life endovascular procedure simulation. The purpose of these models is to provide a tool for preoperative planning and simulation for the endovascular treatment (EVT) of PAD patients.

## 2. Materials and Methods

This study was conducted at the University Hospital of Parma and funded by the Italian Ministry of Health (grant number GR-2019-12369941). Following ethical committee approval, the University Hospital of Parma registered as a manufacturer of custom-made medical devices in the database managed by the Italian Ministry of Health (registration number ITCA01043444). A notification to the Italian Ministry of Health regarding the initiation of the post-market clinical investigation was then submitted. The investigation is conducted in accordance with the EU MDR 2017/745 [21]. According to the risk classification defined in Annex VIII of the MDR, our custom-made 3D-printed vascular models are classified as non-invasive devices, and fall under Class I. This classification denotes the lowest risk category for medical devices, indicating a minimal risk level to patients and users.

From January 2022 to December 2022, ten consecutive patients, aged between 18 and 89 years old, with atherosclerotic disease affecting the aorto-iliac-femoropopliteal district, and presenting with severe claudication or critical limb ischemia (categories 3 to 5, according to Rutherford’s classification) undergoing EVT, were enrolled. Informed consent was obtained from all participants. Exclusion criteria included unavailability of preoperative computed tomography angiography (CTA) for any reason (clinical contraindication, logistical impossibility), need for emergency intervention (lack of time for 3D model printing), or patient refusal to participate in the study.

### 2.1. CTA Image Acquisition and Processing

All of the enrolled patients underwent CTA at the Radiology Unit of the University Hospital of Parma using a 128-slice multidetector CT scanner (SOMATOM Definition Flash, Siemens Healthcare, Forchheim, Germany). Patients were positioned supine, and the imaging protocol included the following three phases: an unenhanced CT scan (SMCT), an angiography phase, and a venous phase (CTV), following conventional parameters.

The examination protocol was as follows: 80 to 120 kVp (adjusted based on the patient’s body mass index), mean X-ray tube current of 240 mAs with automated tube current modulation (CARE Dose 4D), slice thickness/increment of 1.00/0.7 mm, kernel Bv40f/I30f (iterative reconstruction, ADMIRE strength 3), single collimation width of 0.6 mm, rotation time of 0.50 s, and a matrix size of 512 × 512. For contrast enhancement, 70 to 100 mL of contrast medium (Iomeprol, Iomeron 400, Bracco, Milan, Italy), adjusted based on the patient’s body mass index and kilovoltage used, was administered intravenously at a flow rate of 4 to 5 mL/s, followed by a saline chaser (40 to 50 mL) at the same flow rate, using an automatic dual-head pump injector (Stellant, MedRAD, Pittsburgh, PA, USA). A bolus tracking technique (CARE bolus, Siemens, Forchheim, Germany) was used to extend anatomical coverage from the diaphragm to the feet.

**Figure 1:**
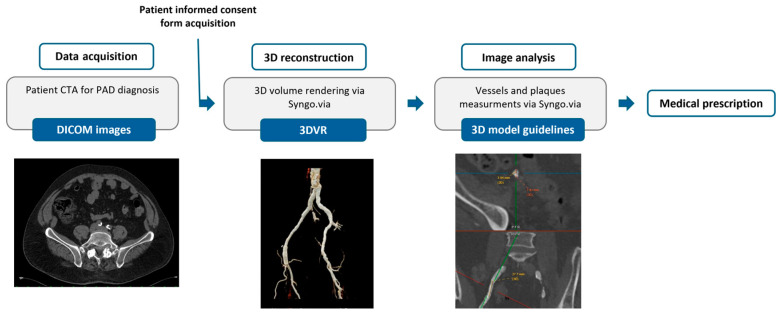
Schematic workflow: from CTA to medical prescription.

Digital Imaging and Communications in Medicine (DICOM) images were acquired and processed to generate 3D reconstructions of the lower-limb arteries. The 3D reconstruction was exclusively based on the angiography phase data, which utilize the contrast agent to enhance blood vessel visibility, ensuring high levels of accuracy. A single researcher conducted the procedure using Syngo.via medical imaging software (Siemens Healthcare GmbH, version VB60A_HF06), specifically utilizing the Syngo.via Cinematic Rendering VRT tool for three-dimensional volume renderings (3DVR). The resulting images were then used for medical prescriptions (Figure 1).

### 2.2. Medical Prescription

According to the EU MDR 2017/745, custom-made devices are medical devices designed exclusively for a particular patient to meet their specific conditions and needs, created based on a written prescription from a qualified healthcare professional [21].

**Figure 2:**
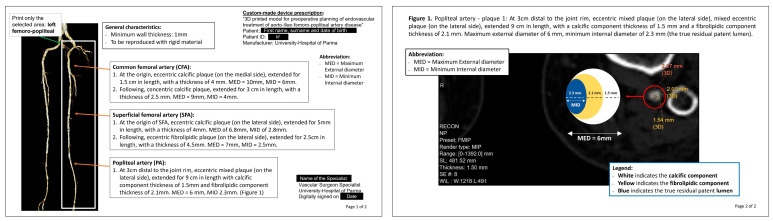
Medical prescription.

In this study, an experienced vascular surgeon examined DICOM images and the 3D reconstruction to formulate a comprehensive medical prescription. The process is illustrated in Figure 1. The dedicated software Syngo.via (Siemens Healthcare GmbH, version VB60A_HF06) was used to accurately analyze the vascular segment requiring treatment. Both the SMCT and the angiography phase were utilized and evaluated section by section. SMCT was particularly useful to discern heavily calcified plaques, and to estimate the residual intravascular lumen. The maximum external diameter and the true residual lumen of the vessel to be treated were measured. A thickness of 1 to 1.5 mm was indicated as the minimum wall thickness to be added to the external wall to enhance the structural integrity of the models, as specified in the medical prescription (Figure 2). Additionally, individual atherosclerotic plaques were characterized based on their length, thickness, composition (calcific, fibrolipidic, or mixed), and position relative to the vessel section. Essential 3D-printed vascular model parameters, including minimum wall thickness and plaque-replication materials, were specified. All of these measurements are particularly important, since the first phases of the printing process require the creation of an .STL file containing only the surface rendering of the internal vessel lumen. This information was detailed on the prescription sheet, along with a schematic representation of the composition and position of each plaque, as shown in Figure 2.

Custom-made devices must also comply with MDR Annex XIII, which includes maintaining specific documentation, such as the “custom-made device statement” [21]. To meet regulatory requirements, a statement for each device detailing the manufacturer, patient, prescribing healthcare professional, and the specific characteristics was edited.

The medical prescription served as a guide for creating the custom-made 3D-printed vascular model.

### 2.3. From DICOM to .STL Files

DICOM images were first imported into OsiriX MD 14.0 (Pixmeo SARL, Geneva, Switzerland), a medical imaging software specifically designed to handle and process DICOM data, facilitating the creation of 3D-printable .STL files. After importing the DICOM series from angiographic data, the relevant dataset was selected based on the vascular region required for procedural planning, as follows: the aortoiliac region for iliac angioplasty and the femoropopliteal or aortoiliac–femoropopliteal area for femoral and popliteal interventions. This selection allowed the model to incorporate sufficient anatomical detail to also simulate crossover techniques when it was needed.

To prepare the 3D model, the region of interest (ROI) tool was initially used to define the area of interest. A circular ROI was manually drawn on a slice where the vascular structure was clearly visible, targeting the region containing the blood vessels. The threshold ROI option was then applied to refine the selection and capture only the relevant anatomy. A minimum threshold of approximately 150 Hounsfield Units (HU) was set to capture softer tissues, while the maximum threshold was adjusted between 600 and 1000 HU. Although a threshold of up to 600 HU typically differentiates vascular structures from surrounding tissues, a higher maximum threshold (up to 1000 HU) was used to include calcified vessels with higher density.

**Figure 3:**
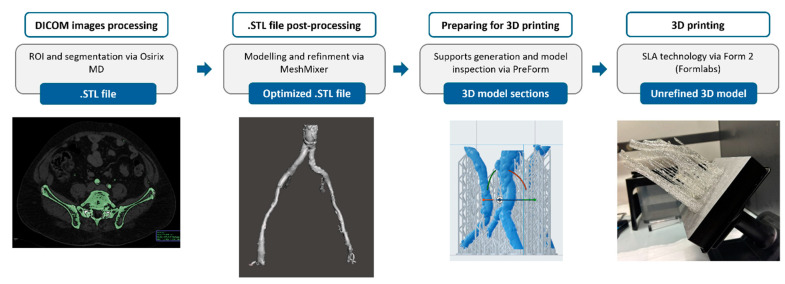
Schematic workflow: from DICOM images to rough 3D-printed model.

To extend the ROI selection across multiple slices, the 3D grow region segmentation tool was utilized. This tool automatically expanded the ROI into adjacent slices, selecting regions that matched the specified threshold settings and ensuring a continuous model of the vascular structures across the dataset (Figure 3). Following segmentation, the 3D surface rendering tool was applied to create a visual model of the segmented vascular area, allowing for preliminary assessment of the 3D structure. However, the high threshold limit also captured dense structures, such as bones, which were segmented alongside the vascular element. Therefore, manual editing was performed using the scissor tool, selectively removing bone structures to ensure only vascular anatomy remained in the final model. This 3D model was then converted into an .STL file format, enabling further refinement and preparation for 3D printing in subsequent software stages.

At this point, the .STL file contains information about the inner vessel lumen (surface rendering based upon the contrast medium in the artery), but not sufficient information about the wall thickness or plaques included. The whole vessel will be re-created during the next phase, following the instructions provided by the above-mentioned medical prescription.

### 2.4. Post-Processing of .STL Files

Meshmixer (San Francisco, CA, USA) is an open-source mesh-editing software also included in the OsiriX MD 14.0 (Pixmeo SARL, Geneva, Switzerland) suite, designed for modifying 3D models and optimizing .STL files for 3D printing. In our methodology, it was used to refine the model before printing, integrating into the same digital model the shape of vessel and atherosclerotic plaques, which were not initially detected during ROI selection, but identified by following the medical prescription instructions. A mesh analysis was first performed to avoid any issues in the .STL file, then rectified using the repair tools. Subsequently, since the vascular region appeared solid, it was hollowed out using the hollow tool. After hollowing, the add tool was used to include the additional plaque shapes, combining them with the vessel model. The editing tools were then employed to further optimize the mesh for 3D printing, such as flattening surfaces and refining details. The optimized .STL file generated in this phase is ready for printing (Figure 3). However, since the vascular 3D model mean volume exceeded the build size of the 3D printer used in our methodology, it had to be separated into multiple parts to avoid the risk of deformation or damage during the printing process. For this reason, the separate shells tool in Meshmixer was used to divide the model into two or three sections, depending on each model’s dimensions.

### 2.5. 3D Printing Phase

The .STL file was 3D-printed using stereolithography (SLA) technology with a Form 2 printer (Formlabs, Somerville, MA, USA) and photo-responsive medical-grade resins. The technical characteristics of the 3D printer are provided in Table 1. Material options included Flexible 80A (Formlabs, Somerville, MA, USA), a translucent elastomer with the flexibility of harder rubber, and Dental LT Clear V2 (Formlabs, Somerville, MA, USA), a rigid transparent material. The choice of the material used to print the custom-made devices was based upon the medical prescription. Dental LT Clear V2 was used for cases where circumferential calcifications covered >80% of the vessel circumference over at least 5 cm in the vascular tract undergoing angioplasty, as the hardness of the material is more representative of reduced vessel deformability and resistance along the lumen trajectory. In contrast, Flexible 80A was chosen for short (<2 cm) and isolated lesions, or soft lesions without calcified components, even if they were longer than 5 cm, as its flexibility more effectively mimics a deformable vessel wall and a smoother lumen trajectory.

The .STL file was uploaded to PreForm 3.28.0 (Formlabs, Somerville, MA, USA), a software designed for preparing 3D models for Formlabs 3D printers. In the setup section, the printer and the material to be used were selected. The necessary support structures were generated to prevent the model from collapsing during printing, with settings customized as needed, as shown in Figure 3. A printability check was conducted, and any required adjustments were made before starting the printing process.

### 2.6. 3D-Printed Model Post-Processing

**Figure 4:**
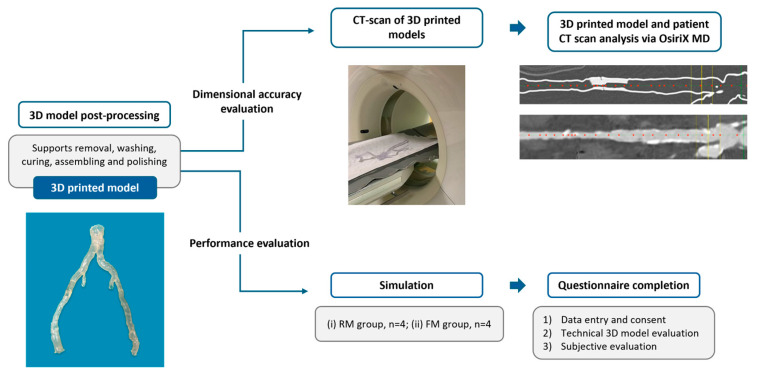
Schematic workflow: from 3D-printed model post-processing to 3D-printed model dimensional accuracy assessment and performance evaluation during simulation.

Once the print was complete, as shown in Figure 3, the 3D hollow vascular model was removed from the build platform, and the support material was manually separated. Subsequently, it underwent washing in two separate containers with isopropyl alcohol (GIP103, Girelli Alcool, Milano, Italy) for approximately 25 min to effectively eliminate any remaining resin residue. Following the cleaning, the model was air-dried and subsequently cured by exposing it to controlled UV light using Form Cure (Formlabs, Somerville, MA, USA). For Dental LT Clear V2 resin, the model was cured at 60 °C for 60 min, while for Flexible 80A, it was cured at 55 °C for 40 min. Then, the sections of the model were glued together, and the model was polished by applying Plastik 70 Kontakt Chemie spray (CRC Industries Europe BVBA, Zele, Belgium) to achieve full transparency, enhancing visualization and contrast during the simulation. Figure 4 shows a 3D-printed model after post-processing, ready for use.

### 2.7. Evaluation of 3D-Printed Model Dimensional Accuracy

To assess the dimensional accuracy of the 3D-printed vascular models, a CT scan of four models was conducted using the same CT device and resolution as those utilized for patients (Figure 4). Subsequently, the 3D model dataset and the corresponding patient CTA data were imported into OsiriX MD 14.0 (Pixmeo SARL, Geneva, Switzerland). The vessel structures of interest in the patient’s CTA datasets were isolated using the segmentation tool. The 3D Volume Rendering (3DVR) modality was used to visualize both the patient region of interest and the 3D-printed model for an initial comparison. Then, a centerline analysis was performed for both datasets using the Vessel Analysis tool, and manually adjusted, if necessary, to ensure accurate alignment with the vessel’s lumen by fine-tuning points along the path (Figure 4). This phase was performed by two authors (AM and GR), and disputes were resolved by the senior author. Once the centerline was confirmed, the inner lumen diameter was measured at 0.5 cm intervals, starting from a defined baseline. The baseline was set at the femoral bifurcation (origin of the superficial femoral artery) for femoropopliteal regions, and at the aortic bifurcation (origin of the common iliac artery) for aorto-iliac regions. Measurements were recorded along the full length of the 3D-printed model scan. Corresponding measurements were taken from the patient’s CT scan along the vascular segment requiring treatment, as prescribed by the medical plan, ensuring alignment with the 3D model’s length. The diameters measured on the CT scans of the printed models were directly compared to those from the original preoperative patient CT scans. This measurement protocol was applied to four patient datasets and their corresponding 3D-printed models (one aorto-iliac and three femoropopliteal), providing a comparative analysis of inner lumen diameter accuracy across both patient and model datasets. The external lumen was excluded from the measurements to avoid potential bias from the added wall thickness, as indicated in the medical prescription.

### 2.8. Evaluation of 3D-Printed Model Performance During Simulation

**Figure 5:**
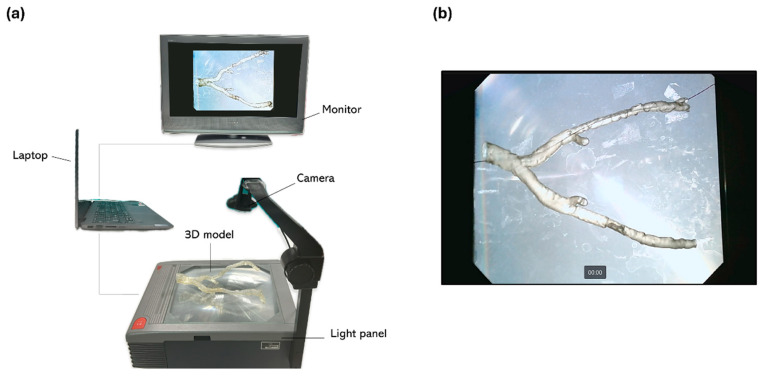
(**a**) Simulation setup: light panel, camera, 3D-printed model, laptop, monitor; (**b**) monitor view.

An in-house, cost-effective simulation setup was created to replicate the angiographic suite environment and fluoroscopy technique. This setup included a light panel, on which the 3D-printed model was placed, with a camera mounted above the panel and connected to a laptop, and a 27-inch monitor for display. The simulation configuration is shown in Figure 5. Five rigid models and five flexible models were used to simulate real-life endovascular procedures. Standard angiography equipment, such as guidewires, catheters, and torques, were employed to navigate the 3D-printed vascular models, with additional materials (angioplasty balloons) available to simulate the treatment of the target lesion. Eight vascular surgeons of the University Hospital of Parma, with varying levels of experience, were included in the study and randomized into the following two groups: the rigid model simulation group (RM group, *n* = 4) and the flexible model simulation group (FM group, *n* = 4). A data entry survey was conducted, and the surgeons provided and signed informed consent to participate in the study. Each surgeon independently performed the simulations on each flexible or rigid model according to their randomization group, and freely choose between a floppy 0.035 guidewire (Terumo, Shibuya City, Tokyo, Japan) or Spartacore 0.014 guidewires (Abbott, Chicago, IL, USA); a Terumo stiff; a 4 Fr BER Tempo Aqua catheter (Cordis, Hialeah, FL, USA), a 4 Fr Vertebral Glidecath catheter (Terumo, Shibuya City, Tokyo, Japan); and 0.035, 0.018, and 0.014 guidewire torques. The evaluation was conducted through two different questionnaires. The first questionnaire (Supplementary Table S1) assessed the technical aspects of the simulation using rigid or flexible 3D-printed models, with each item rated from 1 to 10 points [18]. The second questionnaire (Supplementary Table S2) aimed to assess the physicians’ perceptions of the simulation with 3D-printed models in terms of realism, conceptual impact, and utility for training, pre-procedure rehearsal, and selecting tools and techniques on a Likert scale, a unidimensional psychometric scaling method commonly used in surveys [22]. A schematic of the process described is presented in Figure 4.

### 2.9. Statistical Analysis

The results from the CTA image analysis and both questionnaires were recorded in an Excel spreadsheet (.xls) using Microsoft Excel 365 version 2411 (Microsoft Corporation, Redmond, WA, USA). The technical aspects questionnaire was analyzed using Epi Info 7.2.6.0 (Centers for Disease Control and Prevention, Atlanta, GA, USA), while responses from the physician perception questionnaire were calculated as frequencies in Microsoft Excel.

The minimum internal lumen diameters were measured on both CT scans (the patient’s CT scan and the 3D-printed model CT scan). Dimensional accuracy was assessed by comparing size measurements between the two CT scans using Bland–Altman plots [23].

## 3. Results

### 3.1. Process Time, Materials, and Costs

Ten custom-made 3D-printed vascular models were produced based on ten patients’ CTA data. The time required for processing DICOM images into the .STL file and its post-processing phase varied based on the length of the vascular region of interest and the type of plaques to be reproduced, taking about 7 h per model. Printing time depended on each model’s dimensions and features, averaging 11 h and 25 min (SD = 01:28). Additionally, the model post-processing phase took about 2 h, regardless of the material choice. This resulted in approximately 21 and a half hours to complete the overall process of creating a custom-made 3D-printed vascular model. Data are reported in Table 2.

**Figure 6:**
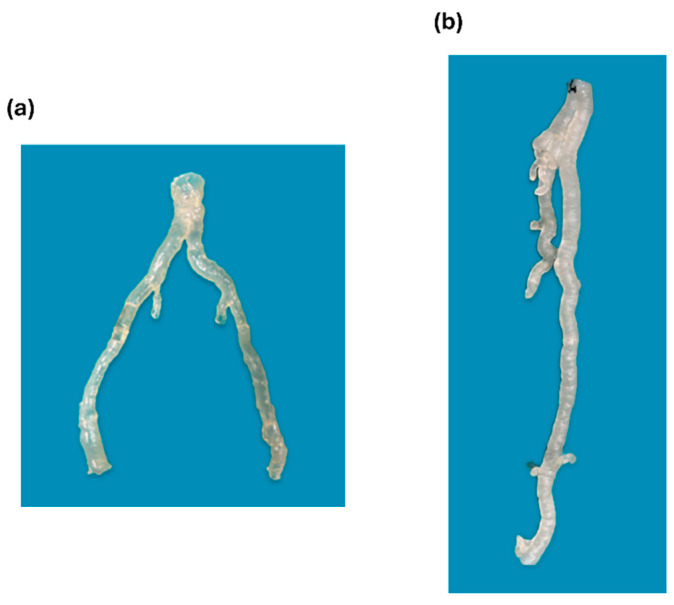
(**a**) Flexible model printed using Flexible 80A; (**b**) rigid model printed using Dental LT Clear V2.

Four models reproduced aorto-iliac lesions and one model produced femoropopliteal lesions; in both cases, they were primarily characterized by fibrolipidic plaques, and were printed using Flexible 80A (Figure 6a). Five models reproduced atherosclerotic femoropopliteal lesions, predominantly featuring circumferential calcific plaques, and were printed using Dental LT Clear V2 (Figure 6b).

The 3D model alone required an average of 96.5 mL of resin (SD = 18.9 mL). Including resin for supports and residual material, the total usage increased to approximately 150 mL per model, regardless of the resin type. At a cost of €426 per liter for Dental LT Clear V2 resin and €243 per liter for Flexible 80A, the approximate cost per model was €65 for Dental LT Clear V2 and €37 for Flexible 80A. In addition, we should consider the costs related to the 3D printer and curing machine, energy supply, disposable materials for printing (build platform and resin tank), and post-processing materials (alcohol), including the operator’s working hours, accounting for about 100 euros per model. Finally, the overall cost to print a flexible, hollow, custom-made 3D-model was approximately 140 euros, and 165 euros for a rigid one. All of the models were successfully printed without any errors occurring during the process. Data are reported in Table 3.

### 3.2. Simulation and Model Evaluation

In the evaluation of patient-specific procedure rehearsals, the rigid and flexible 3D models demonstrated a similar overall performance across key technical aspects. The rigid model performed slightly better in guidewire navigation, advancement through target lesions, and catheter stability, while the flexible model showed advantages in transparency and lesion treatment. However, these differences were not statistically significant. Both models were equally effective in guidewire visualization, and their total scores reflected comparable effectiveness in the rehearsal procedures. Mean values are reported in Table 4.

The subjective evaluation of the simulation indicated positive opinions regarding the realism and utility of the 3D models in patient-specific procedure rehearsals. Overall, both rigid and flexible models were reported to effectively simulate realistic conditions and provide valuable tactile feedback for handling and navigating the vessel with a guidewire or catheter while crossing atheromatous plaques. Their usefulness for training in complex cases of peripheral recanalization was recognized, also assisting physicians in selecting appropriate tools. Table 5 summarizes the responses to the questionnaire.

### 3.3. Assessment of Model Dimensional Accuracy

**Figure 7:**
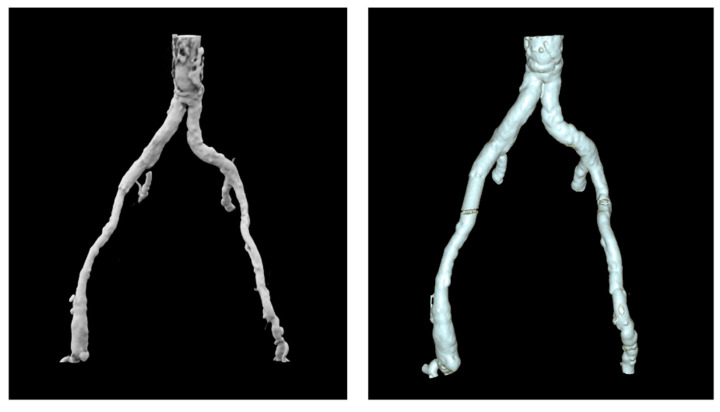
Comparison between the 3DVR of a patient’s CT scan (**left**) and the 3DVR of the respective 3D-printed model’s CT scan (**right**).

The 3DVR tool demonstrated that the 3D-printed models closely resembled the patient’s anatomy, with surface structures accurately mirroring the original vascular region of interest. It also showed the added wall thickness in the 3D-printed model, incorporated as specified in the medical prescription (Figure 7).

**Figure 8:**
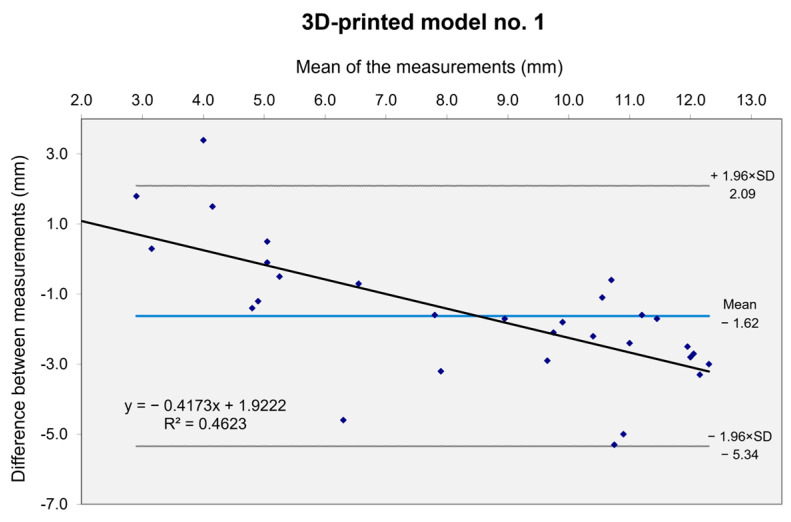

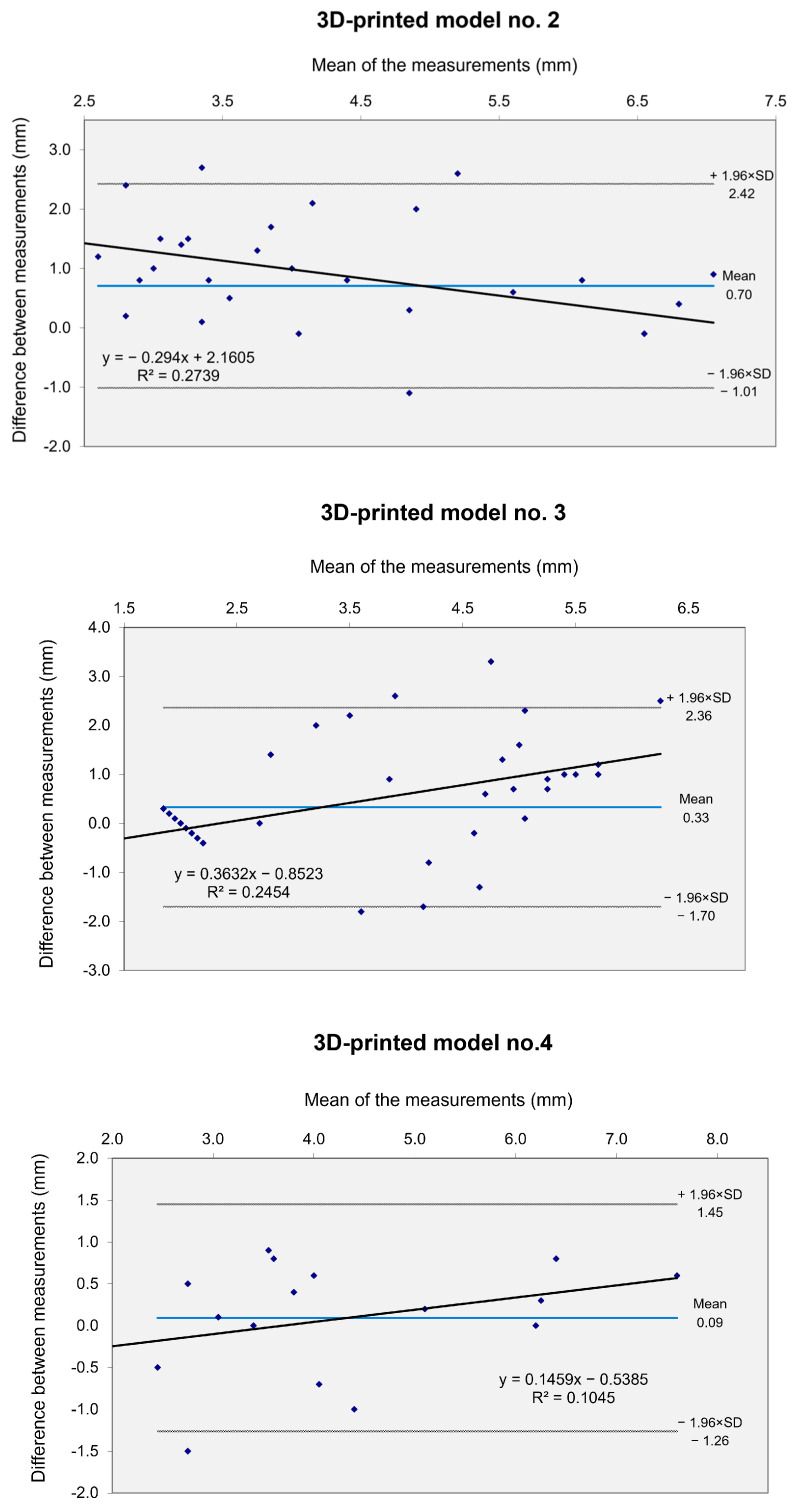
Bland–Altman plots. The *Y*-axis displays the difference between the patient and 3D model CT scan measurements, while the *X*-axis represents the mean of the measurements. Blue dots indicate individual measurement points. The light blue line represents the mean difference, and the gray lines indicate the 95% confidence interval limits (±1.96×SD). The regression line of differences is drawn in black.

The agreement between inner lumen diameter measurements of the patient and 3D-printed model was assessed using Bland–Altman plots (Figure 8), with one plot for each pair, comprising the patient and 3D-printed model CT scan datasets. The first three graphs report data from femoropopliteal regions, whereas the fourth reported data from an aortoiliac region. The mean deviations between the 3D vascular model and patient CT data ranged from −1.62 mm to −0.093 mm, with the majority of the measured values falling within the confidence intervals (CIs), defined as ±1.96 times the SD. Proportional bias was observed in all graphs, with the strongest bias in the first graph (R^2^ = 0.4623) and the weakest in the fourth (R^2^ = 0.1045), indicating slight variation of differences with the mean values. Few outliers were present in each graph, with only 4.3% to 6.9% of data points falling outside the limits of agreement. All 3D-printed vascular models demonstrated reasonable accuracy, with a moderate to weak proportional bias and a correlation trend that aligns closely with the original data.

## 4. Discussion

Endovascular strategies for patients undergoing angioplasty for PAD are typically planned using CTA imaging. However, CTA models often fail to accurately reflect real-life conditions, as vessel anatomy can shift during device advancement, and atherosclerotic plaques may behave unpredictably. Moreover, preoperative CT scans seen on a 2D screen may limit the tri-dimensional understanding of the anatomy and cannot be used for simulation [7]. In contrast, custom-made 3D-printed models may offer more realistic support for precise preoperative planning and device sizing. As an evolving technology, 3D printing is increasingly explored for vascular application [10,24,25,26], emerging as a cost-effective option for cadaveric simulations and virtual/mixed reality (VR/MR) [8,9,10,13]. Mafeld et al. compared a 3D-printed vascular model to VR simulators using Likert scales, demonstrating that the realistic experience provided by both techniques is comparable. However, the current literature does not provide evidence to claim the superiority of 3D-printed models over these established techniques. Studies showed that simulation-based planning and training using 3D-printed models enhance surgical performance, improving intraoperative outcomes [13,18]. However, the literature on 3D-printed vascular models varies in terms of fabrication methods and materials, and only a few studies provide consistent validation of the models or reliable data [13]. The methodology presented in this study addresses the need for a standardized and reproducible fabrication process that enables the generation of high-fidelity, custom-made 3D-printed models.

The fidelity of these models was evaluated by comparing the minimum vascular diameter measurements from the CT scans of the models to those of the patients using Bland–Altman plots. Maximum diameter measurements were excluded to minimize biases arising from the added thickness of the 3D-printed models adjusted during post-processing according to medical prescriptions. The results demonstrated that the 3D-printed models closely replicated real-life measurements, with good agreement between the models and patient data, and only a small percentage of outliers. Two studies in the literature have used similar methods to evaluate the dimensional accuracy of 3D-printed vascular models, though both focused on abdominal aortic aneurysms [19,20]. Nonetheless, Nguyen et al. demonstrated that SLA printers achieved a dimensional error below 1 mm, while Kaschwich et al. used PolyJet technology and Bland–Altman plots to show a strong agreement with the original data.

Accuracy outcomes are influenced by the quality and resolution of the original CT imaging [27,28], as well as the choice of software and the 3D printing technology employed [13,19,20,29]. Our methodology utilized OsiriX MD, a CE-marked commercial medical imaging software (MIS), integrated with Meshmixer, an open-source mesh-editing software (MES). Together, these tools have proven to be effective in balancing precision and accessibility in the creation of anatomically accurate 3D-printed vascular models, as supported by findings from other studies in the literature [29,30,31,32]. However, the use of more advanced commercial MIS and MES, such as those offered by Materialise, which incorporate sophisticated algorithms for segmentation and model optimization, may lead to even higher quality outcomes [15,20,33,34]. As well, the choice of using SLA technology demonstrated a favorable balance between cost and precision, confirming the existing literature findings [20,29,35]. Studies utilizing higher-cost technology printers, such as PolyJet technology, which offers multi-material capabilities, are expected to yield a superior print quality compared to SLA technology printers, which are primarily designed for single-color, single-material prints. However, Nguyen et al. conducted a statistical assessment of aortic 3D-printed models using both printing technologies, and demonstrated that both methods achieved high accuracy, with overall dimensional errors well below 1 mm.

For our purposes, SLA technology achieved a mean printing time of 11 h and 25 min (SD = 01:28), depending on the specific anatomy and on the size of the vascular region of interest. To reduce the active operator time, models were printed overnight, as no intervention is required once printing begins. Post-processing times varied depending on the material used and the model complexity. Support removal was more labor-intensive for 3D models made from rigid resin, while it was easier for flexible ones, and washing took longer for flexible models compared to rigid ones. In both cases, increased model complexity, such as intricate surface structures and internal lumen shapes, extended the time required for manual support removal and washing. Curing times were longer for rigid resin models and shorter for flexible models, though curing required no operator intervention. Vascular anatomical features and surface structures also impacted .STL file processing in the following ways: greater plaque complexity and longer vascular regions increased production time, as replicating more varied and intricate plaques required more time. Likewise, more intricate surface structures required extended refinement for tasks like surface flattening and smoothing. Other studies employing the same or different technologies do not report specific timeframes or delineate the durations associated with individual phases of the production process. Nonetheless, they also showed that the timeframe remains contingent upon the model volume, complexity, and the 3D printing technology utilized [13,14,36,37,38].

The different hardness materials used were both transparent, enabling direct visualization of endovascular materials, such as guidewires and catheters, during the endovascular simulation. The designed simulation setup effectively mimicked the operating room environment and the two-dimensional fluoroscopy images, which is consistent with findings reported in other studies [17,18,37]. In this context, both rigid and flexible models were effective in simulating endovascular procedures. However, while the rigid models performed better in areas such as guidewire navigation and catheter stability, the flexible models excelled in aspects like transparency and lesion treatment. This aligns with other assessments in the literature, suggesting that flexible materials could be better for simulation due to their superior transparency and their ability to predict the behavior of the vessel wall [14,15,19,20,29]. Despite these differences, the total performance scores of the models were comparable, suggesting that both materials can be used effectively. Thus, we recommend using rigid material only in cases of clear and significant circumferential calcifications, where vessel deformability is likely reduced, even with the use of rigid guides. In all other cases, our preferred choice is flexible material.

Positive feedback regarding the models’ realism and tactile sensation highlighted their relevance in mimicking actual procedural conditions. Rigid models, simulating vascular tracts with predominantly circumferential calcifications where vessel deformability is inherently reduced, convey the sensation of the guidewire or catheter encountering higher resistance within the lumen. In contrast, flexible models, simulating vascular tracts with predominantly fibrolipidic plaques, allow for a smoother trajectory, providing a more accurate replication of the dynamics of a deformable vessel wall. These observations align with findings in the literature, demonstrating that 3D models provide tactile feedback that enhances the technical skills that are critical for endovascular procedures [10,13,16,36]. Moreover, the models’ ability to facilitate the selection of appropriate surgical instruments further supports their role as adjuncts in the preoperative setting. Based on our results, we demonstrated that these models are accurate and could play a valuable role in procedural planning and preoperative simulation for EVT. However, their effectiveness should be further validated in clinical settings with real patients. At present, 3D-printed models can be considered valuable tools for simulation-based training in peripheral recanalization procedures, aligning with recent European guidelines that advocate for simulation-based training in vascular surgery [3].

Our methodology considers regulatory compliance and manufacturing standards by adhering to the EU Medical Device Regulation (EU MDR 2017/745), which classifies patient-specific 3D-printed models as custom-made medical devices [21]. Ensuring full compliance with the applicable regulations is crucial, as these regulations establish strict requirements to guarantee the safety, quality, and performance of medical devices. Within this framework, the ability to manufacture medical devices in-house using 3D printing technology and different materials offers a flexible and precise method for creating devices tailored to the clinical case. This approach is particularly advantageous in complex cases where off-the-shelf solutions may fall short. In-house production with SLA technology allowed us to produce high-fidelity 3D-printed models at a relatively low cost. This can lead to substantial savings compared to the outsourcing or purchasing of pre-manufactured devices. Moreover, if we can demonstrate improved patient outcomes, both in terms of reduced treatment times and more efficient use of materials, this approach could lead to even greater cost savings. Particularly for complex cases where conventional solutions might be time-consuming or less effective, in-house production could prove to be both clinically and financially beneficial. In such cases, the investment in this technology could ultimately result in significant long-term savings for healthcare providers, while improving patient care.

The main limitation of this study was the use of a single material, either rigid or flexible, for model fabrication. Material selection was guided by the predominance of circumferential calcified or fibrolipidic plaques in the vascular region undergoing angioplasty, precluding a direct comparison between flexible and rigid materials. Nevertheless, our statistical analysis demonstrated that both materials provided adequate accuracy and enabled effective simulation of key aspects of endovascular procedures. Multi-material 3D printing could enhance model realism by replicating the diverse textures of various tissue types within a single model. However, this approach entails substantially higher costs, both for the materials and the advanced printers required. Another limitation of this study was the small sample size within the flexible and rigid model groups, which may have contributed to the lack of statistical significance in the technical evaluation. Therefore, further studies with larger sample sizes in each group may be needed. Finally, a potential limitation is the influence of human factors on model accuracy. Measurement errors may occur during the creation of the medical prescription, and throughout the plaque reproduction and refinement stages. Higher-cost software that automates these steps could reduce such variability, potentially improving consistency and accuracy. Nonetheless, our methodology focused on balancing reproducibility and affordability to facilitate in-house production, aiming for a cost-effective solution that maintains sufficient fidelity for routine clinical application.

## 5. Conclusions

The proposed methodology balances precision, cost-efficiency, and accessibility, producing accurate and realistic custom-made 3D-printed vascular models that may be useful for preoperative planning and surgical training in PAD interventions. These models offer detailed anatomical representation tailored to the patient, allowing for the rehearsal of complex procedures, and they may aid in device selection for clinical use. By simulating intraoperative conditions, they can improve procedural planning and decision-making, potentially enhancing outcomes in real surgeries. The effectiveness of our method in real-world situations should be confirmed with clinical trials, comparing the use of these 3D models to the standard planning of EVT. This manuscript aims to share our methodology with the scientific community to enable the production of in-house medical devices at very low costs.

## Figures and Tables

**Table 1:** Characteristics of the 3D printer.

Printer	Vendor	Technology	Capabilities	Cost (€)	Build Volume	Layer Thickness	Laser Spot Size
Form 2	Formlabs, Somerville, MA, USA	SLA	Single-color and single-material	3000	145 × 145 × 175 mm	from 0.025 to 0.10 mm	0.14 mm

**Table 2:** Mean time for each phase of 3D-printed model generation process.

Process Phase	Software/Device	Time (h)
From DICOM to .STL file	OsiriX MD 14.0	1
.STL file post-processing	Meshmixer	7
3D model printing	PreForm 3.28.0/Form 2 printer	11.5
3D model post-processing	Form Cure and manual cleaning	2
Overall process	-	21.5

**Table 3:** Characteristics of 3D-printed models.

Printed Model (*n*)	Material Name	Material Characteristics	Femoropopliteal Region (*n*)	Aorto-Iliac Region (*n*)	Cost Per Model (€)
5	Dental LT Clear V2	Rigid, transparent	4	1	165
5	Flexible 80A	Flexible, transparent	1	4	140

**Table 4:** Mean scores for technical evaluation of 3D-printed vascular models (flexible vs. rigid) in patient-specific procedure rehearsal.

Evaluation Step	RM Group (0–10 Points)	FM Group (0–10 Points)	*p*
1. Transparency	8.25	8.75	0.54
2. Guidewire visualization	9.25	9.25	0.87
3. Guidewire navigation	8.75	8	0.29
4. Guidewire advanced through target lesions	9.25	8.75	0.76
5. Stable catheter position	8.75	7.75	0.29
6. Treatment of the lesion	6.5	7.25	0.77
Total score (0–60 points)	50.75	49.75	0.88

Abbreviation: RM = rigid model; FM = flexible model.

**Table 5:** Summaries of the subjective evaluation of patient-specific procedure rehearsal potential responses. DD = DEFINITELY DISAGREE with the statement, D = DISAGREE with the statement, U = UNSURE—you neither agree nor disagree with the statement, A = AGREE with the statement, DA = DEFINITELY AGREE with the statement.

Subjective Evaluation	DA	A	U	D	DD
*n* = 8 (%)					
The model is realistic	7 (87.5)	1 (12.5)			
The simulator provides realistic tactile, “haptic” feedback: guidewire, catheter	4 (50)	4 (50)			
This model is useful for training physicians to perform peripheral recanalization	5 (62.5)	3 (37.5)			
All physicians should train on this model prior performing peripheral recanalization on patients	5 (62.5)	2 (25)	1 (12.5)		
This model is useful for assessment of the skills required to perform peripheral recanalization	6 (75)	2 (25)			
This model is useful to evaluate the tools needed for the “real” case	4 (50)	4 (50)			
This model altered my preconceived concept of the endovascular material		2 (25)	6 (75)		
This simulation is useful to practice the case prior performing the “real” case on the patient	4 (50)	3 (37.5)		1 (12.5)	
I would consider performing a procedure rehearsal before every real case of peripheral recanalization	4 (50)	2 (25)	2 (25)		
I would consider performing a procedure rehearsal only for challenging cases	2 (25)	1 (12.5)	3 (37.5)	2 (25)	

## Data Availability

The original contributions presented in the study are included in the article and Supplementary Materials. Further inquiries can be directed to the corresponding author.

## Supplementary Materials

The following supporting information can be downloaded at: https://www.mdpi.com/article/10.3390/diagnostics15010008/s1, Table S1: Questionnaire for the Technical 3D Model Evaluation of Patient-Specific Procedure Rehearsal; Table S2: Questionnaire for the Validity And Subjective Evaluation of Patient-Specific Procedure Rehearsal Potential.
