# Supplementary material for: In-House Fabrication and Validation of 3D-Printed Custom-Made Medical Devices for Planning and Simulation of Peripheral Endovascular Therapies
Source: Diagnostics (Basel). 2024 Dec 25;15(1):8. doi: 10.3390/diagnostics15010008 (PMC11719810; doi:10.3390/diagnostics15010008)
Supplement: Supplementary file 1 [file diagnostics-15-00008-s001.zip › Supplementary Table S1.pdf]

Technical 3D Model Evaluation of Patient-Specific Procedure Rehearsal

Date of simulation:
Randomization group:
3D Model n°:

Participant Information:

1. **Participant ID:**
2. **Name and Surname:**

Evaluation step	Points (0-10)
1. Transparency	
2. Guidewire visualization	
3. Guidewire navigation	
4. Guidewire advanced through target lesions	
5. Stable catheter position	
6. Treatment of the lesion	
**Total**	
