# Supplementary material for: In-House Fabrication and Validation of 3D-Printed Custom-Made Medical Devices for Planning and Simulation of Peripheral Endovascular Therapies
Source: Diagnostics (Basel). 2024 Dec 25;15(1):8. doi: 10.3390/diagnostics15010008 (PMC11719810; doi:10.3390/diagnostics15010008)
Supplement: Supplementary file 1 [file diagnostics-15-00008-s001.zip › Supplementary Table S2.pdf]

Validity and Subjective Evaluation of Patient-Specific Procedure Rehearsal Potential

Date of simulation:
Randomization group:
3D Model n°:

Participant Information:
1. **Participant ID:**
2. **Name and Surname:**

Circle your answer for each item:

The model is realistic				
1<br>Definitely agree	2<br>Agree	3<br>Unsure	4<br>Disagree	5<br>Definitely disagree
The simulator provides realistic tactile, “haptic” feedback: guidewire, catheter				
1<br>Definitely agree	2<br>Agree	3<br>Unsure	4<br>Disagree	5<br>Definitely disagree
This model is useful for training physicians to perform peripheral recanalization				
1<br>Definitely agree	2<br>Agree	3<br>Unsure	4<br>Disagree	5<br>Definitely disagree
All physicians should train on this model prior performing peripheral recanalization on patients				
1<br>Definitely agree	2<br>Agree	3<br>Unsure	4<br>Disagree	5<br>Definitely disagree
This model is useful for assessment of the skills required to perform peripheral recanalization				
1<br>Definitely agree	2<br>Agree	3<br>Unsure	4<br>Disagree	5<br>Definitely disagree
This model is useful to evaluate the tools needed for the “real” case				
1<br>Definitely agree	2<br>Agree	3<br>Unsure	4<br>Disagree	5<br>Definitely disagree
This model altered my preconceived concept of the endovascular material				
1<br>Definitely agree	2<br>Agree	3<br>Unsure	4<br>Disagree	5<br>Definitely disagree
This simulation is useful to practice the case prior performing the “real” case on the patient				
1<br>Definitely agree	2<br>Agree	3<br>Unsure	4<br>Disagree	5<br>Definitely disagree
I would consider performing a procedure rehearsal before every real case of peripheral recanalization				
1<br>Definitely agree	2<br>Agree	3<br>Unsure	4<br>Disagree	5<br>Definitely disagree
I would consider performing a procedure rehearsal only for challenging cases				
1<br>Definitely agree	2<br>Agree	3<br>Unsure	4<br>Disagree	5<br>Definitely disagree
